# Prediction and consequences of postoperative pancreatitis after pancreaticoduodenectomy

**DOI:** 10.1093/bjsopen/zrac012

**Published:** 2022-04-26

**Authors:** Akseli Bonsdorff, Ilkka Helanterä, Timo Tarvainen, Jukka Sirén, Arto Kokkola, Ville Sallinen

**Affiliations:** 1 Gastroenterological Surgery, Helsinki University Hospital and University of Helsinki, Helsinki, Finland; 2 Transplantation and Liver Surgery, Helsinki University Hospital and University of Helsinki, Helsinki, Finland

## Abstract

**Background:**

Recent studies have suggested postoperative acute pancreatitis (POAP) as a serious complication after pancreaticoduodenectomy (PD) and have speculated on its possible role in the pathogenesis of postoperative pancreatic fistula (POPF). This study aimed to assess the impact of POAP on post-PD outcomes and fistula risk score (FRS) performance in predicting POAP.

**Methods:**

All PDs at Helsinki University Hospital between 2013 and 2020 were analysed. POAP was defined as a plasma amylase activity greater than the normal upper limit on postoperative day (POD) 1 and stratified as clinically relevant (CR)-POAP once C-reactive protein (CRP) reached or exceeded 180 mg/l, and non-CR-POAP once CRP was less than 180 mg/l on POD 2. The Comprehensive Complication Index (CCI) was used to assess total postoperative morbidity. Different FRSs were assessed using receiver operating characteristic curves.

**Results:**

Of the 508 patients included, POAP occurred in 202 (39.8 per cent) patients, of whom 91 (17.9 per cent) had CR-POAP. The incidence of CR-POPF was 12.6 per cent (64 patients). Patients with non-CR-POAP had a similar morbidity to patients with no POAP (median CCI score 24.2 *versus* 22.6; *P* = 0.142), while CCI score was significantly higher (37.2) in patients with CR-POAP (*P* < 0.001). CR-POAP was associated with increased rates of CR-POPF, delayed gastric emptying, haemorrhage, and bile leak, while non-CR-POAP was associated only with CR-POPF. Ninety-day mortality was 1.6 per cent, 0.9 per cent, and 3.3 per cent in patients with no-POAP, non-CR-POAP, and CR-POAP, respectively. Updated alternative FRS showed the best performance in predicting CR-POAP (area under the curve 0.834).

**Conclusion:**

CR-POAP was associated with a higher CCI score, suggesting CR-POAP as a distinct entity from non-CR-POAP. FRSs can be used to assess the risk of CR-POAP.

## Teaser

In this retrospective cohort study, 18 per cent of 508 patients undergoing pancreaticoduodenectomy had clinically relevant postoperative pancreatitis, which increased the overall morbidity of these patients. The available and validated fistula risk scores can also be used to predict the risk of postoperative pancreatitis.

## Introduction

Postoperative acute pancreatitis (POAP) after pancreaticoduodenectomy (PD) has recently been a topic of eager discussion among pancreatic surgeons. Characterized by postoperative plasma hyperamylasaemia, POAP is considered the manifestation of an acute inflammatory process of the pancreatic remnant, possibly due to local hypoperfusion or pancreatic microtrauma during the surgical procedure^1,2^. POAP has been speculated to have a detrimental effect on the healing of pancreatic anastomosis, potentially triggering more severe morbidity, including the pathogenesis of postoperative pancreatic fistula (POPF)^3,4^.

While some studies confirmed the association of POAP with worse postoperative outcomes, the lack of a uniform definition has prevented POAP from being demonstrated as a specific postoperative complication rather than just a biochemical manifestation of POPF^4–9^. Connor’s definition for POAP^1^ is the most widely used and considers a cutoff for serum pancreatic enzymes greater than the upper limit of normal eventually combined with an increase in C-reactive protein (CRP) to characterize clinically relevant (CR)-POAP. The threshold for plasma enzymes of revised Atlanta criteria^10^ has also been used in the recent literature, as some studies have questioned Connor’s definition for including many patients without clinical signs of pancreatitis, thereby losing specificity^4,11^. However, most have primarily focused on POAP, while CR-POAP, potentially with higher specificity, has been less thoroughly investigated^12^.

Preoperative risk scores, such as Fistula Risk Score (FRS), have been formulated for predicting CR-POPF^13–15^ but never assessed for POAP prediction. Single studies have demonstrated non-dilated main pancreatic duct and high acinar cell density at the resection line to be significant risk factors for POAP, and Partelli *et al*. have shown that FRS correlates positively with acinar cell density^3,16,17^.

This study aimed to validate the effect of CR-POAP on postoperative outcomes after PD by applying the Comprehensive Complication Index (CCI) to compare individual outcomes^18^. In addition, CR-POAP risk factors were investigated to assess the ability of previously validated risk scores to predict the occurrence of CR-POAP.

## Methods

### Patient inclusion and data collection

This study was approved by our institutional research committee (Helsinki University Hospital/115/2020). Data from all patients undergoing PD from 1 January 2013 to 30 October 2020 were retrospectively collected and analysed. All procedures were carried out at the Helsinki University Hospital, an academic teaching hospital functioning as a secondary and tertiary referral centre. Collected data included demographics, operative details, postoperative data, tumour histology, and follow-up. The last date of follow-up was defined as any contact with health care. Preoperative comorbidities were recorded and rated according to the Charlson Comorbidity Index^19^.

### Operative details

Both Whipple and pylorus-preserving PDs were included in the study. Pancreaticojejunostomy was performed in a duct-to-mucosa fashion with two-layered anastomosis in all patients. Two intra-abdominal passive 24 Fr drains were always placed, and drain removal was based on low output and drain fluid amylase activity at postoperative days (POD) 1 to 3. The perioperative administration of somatostatin analogue, mainly pasireotide, up to POD 6 was used selectively for patients with a high-risk pancreas (soft texture and non-dilated main pancreatic duct). In addition, some of the patients were included between 2016 and 2018 in a randomized controlled trial comparing perioperative hydrocortisone to pasireotide^20^.

### Postoperative data

All postoperative complications up to POD 30 were collected and classified according to the Clavien-Dindo classification (CD)^21^. Cumulative postoperative morbidity was assessed using the CD-based CCI^18^. PD-specific complications, including POPF^22^, postpancreatectomy haemorrhage^23^, delayed gastric emptying^24^, chyle leak^25^, and bile leak^26^, were defined according to the International Study Group for Pancreatic Surgery (ISGPS) and the International Study Group of Liver Surgery guidelines. Length of stay was defined as the POD on which patient was discharged after the index operation. Readmission was defined as a new hospital admission before POD 30. Mortality was considered to be postoperative if occurring before POD 90. A CCI score of 33.7 or more (which equals the total cumulative morbidity of one reoperation under general anaesthesia) was used to define patients with high postoperative morbidity, and a CCI score less than 12.3 (which results from two CD I complications) was used to define patients with low morbidity.

Laboratory variables included plasma amylase, drain fluid amylase, and plasma CRP. Owing to the lack of a widely accepted definition for POAP, Connor’s definition of plasma amylase activity greater than the upper limit of normal (ULN) was applied, and a POD2 CRP level of 180 mg/l or greater was used for defining CR-POAP^1^. Owing to the mutual inclusivity of POAP and CR-POAP, a non-CR-POAP was defined as POD1 plasma amylase greater than the ULN but POD2 CRP < 180 mg/l. Patients with plasma amylase in the standard range or below assessed on a POD1, regardless of CRP, were classified as having no POAP.

The institutional ULN for plasma pancreatic amylase was 65 U/l. Plasma amylase levels were usually measured on POD1 and POD3, but owing to the long study period and slightly differing postoperative policies, some patients had missing measurements on these days and were excluded from the study. Patients with missing POD2 CRP measurements were also excluded.

To assess the risk of POPF and investigate the risk factors for POAP, different FRS were applied. The original FRS^27^, alternative-FRS^14^, and updated alternative-FRS^15^ were included. According to pancreatic texture, tumour histology, main pancreatic duct diameter, and intraoperative blood loss, FRS rates patients on a scale from 0 to 10, where higher grades represent higher risks for POPF. The alternative-FRS considers the pancreatic texture, main pancreatic duct diameter, and BMI, while the Updated alternative-FRS adds the effect of sex to the alternative-FRS. Also, a recent risk stratification matrice for POPF, based solely on pancreatic texture and main pancreatic duct diameter, from ISGPS was included for validation and POAP risk assessment^28^.

### Statistics

Continuous variables are reported as median and interquartile range (i.q.r.), and categorical variables as frequencies and proportions (per cent). Differences between continuous variables were assessed using the Mann–Whitney U test; for categorical variables, differences were assessed using Fisher’s exact test or the χ^2^ test. Receiver operating characteristic (ROC) curves were used to analyse the association of FRS and the occurrence of CR-POAP and CR-POPF, and the association of plasma amylase values and morbidity. Logistic binary regression was performed to assess the potential risk factors for CR-POAP. Fisher’s exact test was used to assess associations in univariable analysis. An unadjusted two-sided *P* value of less than 0.10 was required for inclusion in the multivariable analysis, and a two-sided *P* value of less than 0.05 in multivariable analysis was used to identify independent risk factors. In general, a two-sided *P* value of less than 0.05 was considered to be statistically significant. Statistical analysis was performed using SPSS 27.0 software (SPSS 27.0 for Macintosh, IBM, Armonk, NY, USA).

## Results

### Patient demographics

A total of 614 patients underwent PD during the study period. After excluding patients with missing POD 1 plasma amylase and POD 2 CRP values, a total of 508 (82.7 per cent) patients were included in the analyses. Basic demographics are reported in *Table 1*.

**Table 1:** Patient demographics, perioperative data, and pathology of 508 patients undergoing pancreaticoduodenectomy

	*n* (%) or median (i.q.r.)
**Age (years)**	68 (61–73)
**Sex ratio (M : F)**	277 : 231 (54.5 : 45.5)
**BMI (kg/m^2^)** *	25.5 (23.0–28.1)
**Charlson Comorbidity -index**	2 (2–3)
**Comorbidities**	
MI	35 (6.9)
CHF	24 (4.7)
Peripheral vascular disease	30 (5.9)
CVA or TIA	25 (4.9)
Hemiplegia	1 (0.2)
Dementia	5 (1.0)
COPD	68 (13.4)
Connective tissue disease	14 (2.8)
Peptic ulcer disease	5 (1.0)
DM without end-organ complications	122 (24.0)
DM with end-organ complications	6 (1.2)
Moderate-to-severe CKD	15 (3.0)
Liver disease	7 (1.4)
Leukaemia	2 (0.4)
Lymphoma	6 (1.2)
**Preoperative medication**	
Anticoagulation	61 (12.0)
Immunosuppression	9 (1.8)
Corticosteroid	26 (5.1)
**Preoperative ERCP**	368 (72.4)
**Neoadjuvant therapy**	106 (20.9)
**Venous resection**	126 (24.8)
**Arterial resection**	15 (3.0)
**Pancreatic texture**	
Soft	259 (51.0)
Non-soft	249 (49.0)
**Main pancreatic duct diameter (mm)** *	
>3	265 (52.2)
≤ 3	242 (47.8)
**Estimated blood loss (ml)**	650 (400–1100)
**Pathology**	
PDAC	269 (53.0)
Ampullary adenocarcinoma	52 (10.2)
Cholangiocarcinoma	46 (9.1)
Neuroendocrine tumour	22 (4.3)
IPMN	21 (4.1)
Duodenal adenocarcinoma	16 (3.1)
Other	82 (16.1)

* Data not available on all patients: BMI available in 507/508 patients; main pancreatic duct diameter available in 507/508 patients. i.q.r., interquartile range; M, male; F, female; MI, myocardial infarction; CHF, congestive heart failure; CVA, cerebrovascular accident; TIA, transient ischaemic attack; COPD, chronic obstructive pulmonary disease; DM, diabetes mellitus; CKD, chronic kidney disease; ERCP, endoscopic retrograde cholangiopancreatography; PDAC, pancreatic ductal adenocarcinoma; IPMN, intraductal papillary mucinous neoplasm.

### Incidence and outcomes of POAP and CR-POAP

Different cutoffs were investigated for POD1 plasma amylase in predicting CR-POPF and a CCI-score of 33.7 or more. The area under the curve (AUC) value for POD 1 plasma amylase was 0.86 for predicting CR-POPF and 0.65 for a CCI of 33.7 or more. Connor’s POAP had AUC values of 0.79 for CR-POPF and 0.61 for a CCI of 33.7 or more. A cutoff of 1.5 times the ULN for plasma amylase performed the best with AUC values of 0.80 and 0.62. Given the slightest difference from Connor’s cutoff, further analyses maintained Connor’s definition (*Fig. S1*).

POAP occurred in 202 (39.8 per cent) patients: 91 with CR-POAP (17.9 per cent, or 45.0 per cent of patients with POAP) and 111 with non-CR-POAP (21.9 per cent, or 55.0 per cent of patients with POAP). CR-POPF occurred in 64 (12.6 per cent) patients. The median CCI score of the whole cohort was 24.2 (i.q.r. 15.0 to 34.6).

Postoperative outcomes stratified by the occurrence of CR-POAP are reported in *Table 2*. CCI score was significantly higher in patients with CR-POAP compared with non-CR-POAP (37.2 *versus* 24.2; *P* < 0.001) or no-POAP patients (37.2 *versus* 22.6; *P* < 0.001). No difference in CCI score was highlighted between non-CR-POAP and no-POAP groups (24.2 *versus* 22.6; *P* = 0.142). All clinically relevant complications—except for chyle leak—occurred significantly more in patients with CR-POAP than those with non-CR-POAP or no-POAP (*Table 2*). Only six (6.6 per cent) patients with CR-POAP had a low postoperative morbidity (CCI score < 12.3) (*Table 2*). The rate of POPF (any grade) and CR-POPF progressively increased from no-POAP patients to the non-CR-POAP and up to the CR-POAP-group: 6.2 per cent *versus* 59.5 per cent *versus* 76.2 per cent (*P* < 0.001) for any grade POPF, and 2.0 per cent *versus* 10.8 per cent *versus* 50.5 per cent (*P* < 0.001) for CR-POPF, respectively. Length of stay was significantly longer in patients with CR-POAP compared with patients with non-CR-POAP or no POAP (13 *versus* 9 *versus* 9; *P* < 0.001). Postoperative mortality or readmission did not differ between the groups (*Table 2*).

**Table 2:** Postoperative outcomes of 508 patients undergoing pancreaticoduodenectomy, stratified by no postoperative acute pancreatitis (POAP), non-clinically relevant (CR)-POAP, and CR-POAP

	No POAP (*n* = 306)	Non-CR-POAP (*n* = 111)	*P* value (no *versus* non-CR-POAP)	CR-POAP (*n* = 91)	*P* value (no *versus* CR-POAP)	*P* value (non-CR- *versus* CR-POAP)
**Continuous variables, median (i.q.r.)**						
CCI score	22.6 (12.2–32.0)	24.2 (15.0–33.1)	0.142	37.2 (30.8–47.6)	<0.001	<0.001
Drain fluid amylase on POD 3 (U/l)	20 (10–67)	590 (150–1600)	<0.001	1300 (390–3300)	<0.001	<0.001
Length of initial hospital stay (days)	9 (7–12)	9 (8–13)	0.063	13 (9–18)	<0.001	<0.001
**Categorical variables, *n* (%)**						
CCI score ≥ 33.7* (*n* = 141)	62 (20.3)	23 (20.7)	0.918	56 (61.5)	<0.001	<0.001
CCI score < 12.3† (*n* = 153)	123 (29.9)	24 (21.6)	0.087	6 (6.6)	<0.001	0.003
POPF, grades BL, B, and C (*n* = 155)	19 (6.2)	66 (59.5)	<0.001	70 (76.9)	<0.001	0.010
CR-POPF (*n* = 64)	6 (2.0)	12 (10.8)	<0.001	46 (50.5)	<0.001	<0.001
CR-DGE (*n* = 88)	49 (16.0)	15 (13.5)	0.531	24 (26.4)	0.025	0.031
CR-PPH (*n* = 20)	9 (2.9)	3 (2.7)	0.898	8 (8.8)	0.016	0.068
Chyle leak (*n* = 87)	55 (18.0)	25 (22.5)	0.297	7 (7.7)	0.018	0.006
CR-bile leak (*n* = 19)	8 (2.6)	3 (2.7)	0.960	8 (8.8)	0.009	0.068
Readmission (*n* = 56)	31 (10.1)	12 (10.8)	0.856	13 (14.2)	0.342	0.523
90-day mortality (*n* = 9)	5 (1.6)	1 (0.9)	1.00	3 (3.3)	0.391	0.329

Data are median (interquartile range) or *n* (%). *Equals the total cumulative morbidity of one reoperation under general anaesthesia (i.e. equal to high postoperative morbidity). †Equals the total cumulative morbidity of two Clavien-Dindo I complications (i.e. equal to low postoperative morbidity). i.q.r., interquartile range; CCI, Comprehensive Complication Index; POD, postoperative day; POPF, postoperative pancreatic fistula; DGE, delayed gastric emptying; PPH, post pancreatectomy haemorrhage.

CR-POAP had a 61.5 per cent positive predictive value (PPV), 79.6 per cent negative predictive value (NPV), 39.7 per cent sensitivity, 90.4 per cent specificity, and 76.4 per cent accuracy in predicting a CCI of 33.7 or more, and 50.5 per cent PPV, 95.7 per cent NPV, 71.9 per cent sensitivity, 89.9 per cent specificity, and 87.6 per cent accuracy in predicting CR-POPF. In contrast, POAP—as defined by Connor—had a 39.1 per cent PPV, 79.7 per cent NPV, 56.0 per cent sensitivity, 66.5 per cent specificity, and 63.6 per cent accuracy in predicting a CCI of 33.7 or more, and 28.7 per cent PPV, 98.0 per cent NPV, 90.7 per cent sensitivity, 67.6 per cent specificity, and 70.5 per cent accuracy in predicting CR-POPF.

In patients with an elevated POD 2 CRP, the possible increased value of POAP diagnosis in predicting morbidity is reported in *Table 3*. CCI score was significantly higher in patients with CR-POAP compared with patients with no POAP but a CRP of 180 or more on POD2 (*n* = 75; CCI 37.2 *versus* 29.6 (*P* < 0.001)). The PPV of an exclusively elevated CRP on POD 2 in predicting a CCI of 33.7 or more was 27.0 per cent *versus* 61.5 per cent of CR-POAP. Multivariable analysis on predictors of a CCI of 33.7 or more identified CR-POAP (odds ratio (o.r.) 2.9, 95 per cent confidence interval (c.i.) 1.57 to 5.35), CR-POPF (o.r. 3.81, 95 per cent c.i. 1.93 to 7.53), and BMI (o.r. 1.07 per unit of increase, 95 per cent c.i. 1.02 to 1.13) as independent risk factors of high postoperative morbidity (*Table S1*).

**Table 3:** The increased value of postoperative acute pancreatitis (POAP) diagnosis on patients with postoperative day (POD) 2 C-reactive protein (CRP) of 180 mg/l or higher

	POD 2 CRP ≥ 180 mg/l, normal amylase (*n* = 75)	CR-POAP (*n* = 91)	*P* value
**CCI score**	29.6 (20.9–37.2)	37.2 (30.8–47.6)	<0.001
**Drain fluid amylase (U/l)**	48 (16–190)	1300 (390–3300)	<0.001
**Length of stay (days)**	11 (8–14)	13 (9–18)	0.005
CCI score ≥ 33.7*	20 (27.0)	56 (61.5)	<0.001
**POPF, grades BL, B, and C**	13 (17.3)	70 (76.9)	<0.001
**CR-POPF**	4 (5.3)	46 (50.5)	<0.001

Data are median (interquartile range) or *n* (%). *Equals the total cumulative morbidity of one reoperation under general anaesthesia. CCI, Comprehensive Complication Index; POPF, postoperative pancreatic fistula; CR, clinically relevant.

### Prediction of CR-POAP

Uni- and multivariable analyses investigating predictors of CR-POAP are reported in *Table 4*. In a multivariable model, male sex (o.r. 2.47), soft pancreatic texture (o.r. 7.11), BMI (o.r. 1.14 for one unit increase), and main pancreatic duct diameter (o.r. 0.78 for one unit increase) were deemed independent risk factors for CR-POAP. A model containing all the independent risk factors had an AUC value of 0.866 (95 per cent c.i. 0.830 to 0.902) in predicting CR-POAP.

**Table 4:** Results of univariable and multivariable analyses on predictors for clinically relevant postoperative acute pancreatitis (CR-POAP) in 508 patients undergoing pancreaticoduodenectomy

	Univariable analysis of risk factors for CR-POAP			Multivariable analysis of risk factors for CR-POAP		
	CR-POAP (*n* = 91)	No CR-POAP (*n* = 417)	*P* value	o.r.	95% c.i.	*P* value
**Continuous variables**						
Age (years)	67 (58–74)	68 (61–73)	0.498			
BMI (kg/m^2^)	26.93 (24.90–29.64)	25.24 (22.53–27.47)	< 0.001	1.14	1.07–1.22	< 0.001
				Unit of increase: 1 kg/m^2^		
Charlson index	3 (2–4)	2 (2–3)	0.224			
Estimated blood loss (ml)	600 (450–1000)	700 (400–1200)	0.573			
Main pancreatic duct diameter (mm)	2 (2–3)	4 (3–6)	< 0.001	0.78	0.66–0.93	0.006
				Unit of increase: 1 mm		
**Categorical variables**						
Sex						
Male	63 (69.2)	214 (51.3)	0.002	2.47	1.37–4.43	0.003
Female	28 (30.8)	203 (48.7)		Ref.		
Neoadjuvant therapy	6 (6.6)	100 (24.0)	< 0.001	0.65	0.23–1.82	0.414
Preoperative ERCP	60 (65.9)	308 (74.0)	0.121			
Venous resection	7 (7.7)	119 (28.5)	< 0.001	2.28	0.88–5.91	0.091
Soft pancreatic texture	85 (93.4)	174 (41.7)	< 0.001	7.11	2.70–18.71	< 0.001
Tumour histology						
PDAC	21 (23.1)	248 (59.8)	< 0.001	1.03	0.39–2.70	0.954
IPMN or MCN	5 (5.5)	30 (7.2)	0.655			
NET	4 (4.4)	18 (4.3)	1.00			
Extrapancreatic malignancies (cholangioCA, papillaryCA, duodenalCA)	39 (42.9)	75 (18.0)	< 0.001	2.31	0.93–5.73	0.071
Other	22 (24.1)	46 (11.0)	< 0.001	1.85	0.69–4.95	0.223

Data are median (interquartile range) or *n* (%). o.r., odds ratio; c.i., confidence interval; ERCP, endoscopic retrograde cholangiopancreatography; PDAC, pancreatic ductal adenocarcinoma; IPMN, intraductal mucinous papillary neoplasm; MCN, mucinous cystic neoplasm; NET, neuroendocrine tumour; CA, carcinoma.

**Fig. 1:**
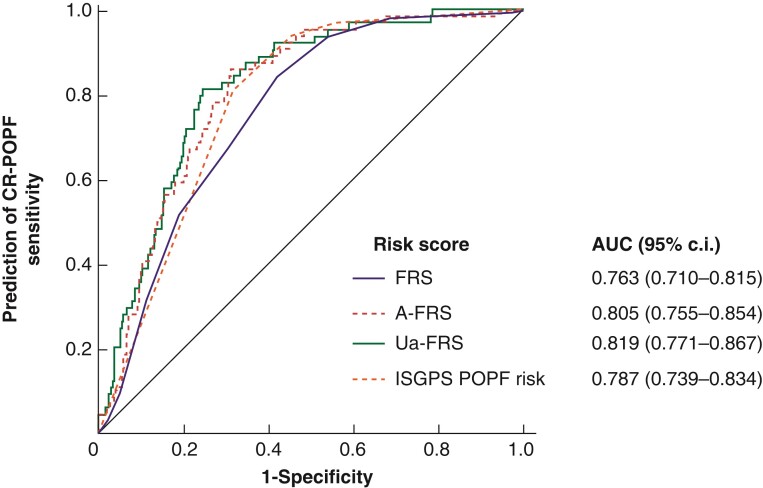
Results of the receiver operating characteristic curve analyses in predicting clinically relevant postoperative pancreatic fistula after pancreaticoduodenectomy

ROC curves assessing the diagnostic performance of different FRS for predicting CR-POPF are displayed in *Fig. 1*. The updated alternative-FRS showed the highest AUC value (0.819), followed by the alternative-FRS (0.805), the novel ISGPS POPF risk stratification (0.787)^28^, and the original FRS (0.763).

Results of the receiver operating characteristic curve analyses of Fistula Risk Score (FRS), alternative FRS (A-FRS), updated alternative FRS (UA-FRS), and International Study Group for Pancreatic Surgery (ISGPS) postoperative pancreatic fistula (POPF) risk matrice in predicting clinically relevant POPF (CR-POPF) after pancreaticoduodenectomy. AUC, area under the curve; c.i., confidence interval.

**Fig. 2:**
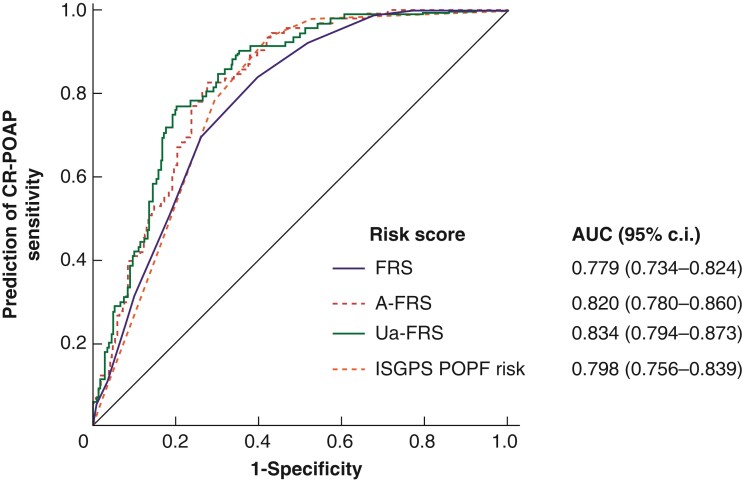
Results of the receiver operating characteristic curve analyses in predicting clinically relevant postoperative pancreatitis after pancreaticoduodenectomy

Similar curves were plotted for predicting CR-POAP and are reported in *Fig. 2*. All risk scores performed better in predicting CR-POAP than CR-POPF. The updated alternative-FRS showed the best performance in predicting CR-POAP, with the highest AUC value of 0.834.

Results of the receiver operating characteristic curve analyses of Fistula Risk Score (FRS), alternative FRS (A-FRS), updated alternative FRS (UA-FRS), and International Study Group for Pancreatic Surgery (ISGPS) postoperative pancreatic fistula (POPF) risk matrice in predicting clinically relevant postoperative pancreatitis (CR-POAP) after pancreaticoduodenectomy.

## Discussion

CR-POAP carries serious risks for subsequent morbidity, as it has been associated with significantly more cumulative postoperative complications than non-CR-POAP or no POAP at all. These translate into a longer initial hospital stay, while patients with non-CR-POAP had a similar length of stay than those with no POAP. Multivariable analysis determined CR-POAP to be an independent risk factor for high postoperative morbidity (CCI of 33.7 or more), regardless of the occurrence of CR-POPF.

Interestingly, previously established risk scores for CR-POPF seemed to predict CR-POAP with even higher accuracy. The updated alternative FRS, which considers pancreatic texture, main pancreatic duct diameter, sex, and BMI, showed the best performance in predicting CR-POAP.

POAP is still a controversial topic. The lack of a validated uniform definition and variability in metrics between previous studies have limited the production of high-quality evidence and meta-analyses^5^. For these reasons, this study focused on finding and validating risk factors for POAP defined according to the most frequently applied definition proposed by Connor^1^. A considerable limitation of Connor’s definition is the low specificity for detecting complicated postoperative courses. The occurrence of Connor’s POAP has been reported to be high, ranging from 39.8 per cent in this study cohort to as high as 64 per cent^4^, confirming that POAP frequently occurs after PD, and a proportion of patients do not evolve towards more severe clinical states.

An elevation of CRP over 180 mg/l on POD 2 was therefore included to characterize clinically relevant POAP with significantly better PPV, specificity, and accuracy than Connor’s POAP in predicting complications. The clinical relevance of POAP based on alterations in the clinical course, mirroring the ISGPS classification of CR-POPF, was also proposed, but an early biochemical alteration in inflammatory markers may be crucial for risk stratification already on POD 2. CR-POAP seems to identify patients at high risk for further morbidity, who could be the target of therapeutic strategies, such as continuing pasireotide administration and maintaining intra-abdominal drains.

Most of the previous studies have focused mainly on the incidence and outcomes of POAP^2,3,8^, while the few evaluating CR-POAP applied inconsistent definitions^6,9,11,12,29^. Loos *et al*. reported that a POD 2 CRP value greater than 135 mg/l along with a POD1 plasma amylase value of three times the ULN was predictive of postoperative pancreatitis verified at CT^11^. Instead of a POD 2 assessment as proposed by Connor, Ikenaga *et al*. used POD3 CRP values to define CR-POAP, showing its association with postoperative complications, especially CR-POPF^9^. Partelli *et al*. used the appropriate Connor’s definition confirming CR-POAP as a significant risk factor for CR-POPF^12^.

The outcomes of patients with elevated plasma amylase alone (non-CR-POAP) did not significantly differ from patients without POAP, in contrast to the poor outcomes of patients with elevated amylase and CRP, namely CR-POAP. Non-CR-POAP still carries a higher risk for CR-POPF than no POAP, but that proportion was lower (10.8 per cent) than patients with CR-POAP (50.5 per cent). These results suggested that CR-POPF may occur independently or be the consequence of CR-POAP. Multivariable analysis of predictors for high postoperative morbidity also showed that CR-POAP was independently associated with higher postoperative morbidity regardless of the occurrence of CR-POPF. It has been speculated that CR-POPF might be a possible cause of non-CR-POAP, as the leakage of enzyme-rich fluid from the pancreatic stump could result in fluid absorption and increased plasma amylase activity. However, these hypotheses have not been confirmed, noting that CR-POAP is an early postoperative phenomenon that could drive, but is not limited to driving, the development of CR-POPF and other complications. Non-CR-POAP could be analogous to the nomenclature of ‘biochemical leak’ in the POPF definition^22^ as a biochemical sign of pancreatic tissue irritation without clinically relevant changes in outcomes.

While several recent studies have addressed the growing interest in defining POAP, its predictors have been less well investigated. A systematic review summarized factors associated with postoperative hyperamylasaemia (considering various definitions) in studies published before June 2019^5^. Soft pancreatic texture, small main pancreatic duct, and non-PDAC pathology were predisposing factors, while exocrine insufficiency, neoadjuvant therapy, and additional resection of the pancreatic stump were protective factors for postoperative hyperamylasaemia. A study by Chen *et al.* specifically assessed the predictors for POAP and reported, in addition, female sex, normal bilirubin levels, and robotic surgery among the risk factors^17^. However, predictors for CR-POAP have never been examined. Multivariable analysis showed that male sex, high BMI, small main pancreatic duct, and soft pancreatic texture were independent risk factors for CR-POAP, with the highest risk attributed to a soft pancreatic texture (o.r. 7.11).

The FRS^27^ was established in 2013 to predict CR-POPF, and updated versions have been published and validated since then. The updated alternative FRS^15^ is the most recent and achieved the best prediction of CR-POPF in this study. Interestingly, all the risk scores predicted CR-POAP better than CR-POPF, including the updated alternative FRS, which showed the best predicting performance for CR-POAP. As the risk factors of CR-POAP and CR-POPF overlap, we can speculate that these two entities eventually share the same origin. Whether it is pancreatitis that precedes fistula or a currently unknown entity that precedes them both cannot be inferred from this study. However, well-established FRS can be used to predict CR-POAP accurately, without the need for additional specific risk scores.

The present study has some limitations in addition to its observational nature. Data on plasma amylase and CRP levels were missing in approximately 15 per cent of patients, and were consequently excluded from analyses, questioning whether the final cohort reflects the entire population. In addition, it might be argued that defining CR-POAP with CRP levels is disingenuous as such an increase may result from the expected inflammatory process. However, POD 2 is early enough to have reasonable clinical applicability as most severe complications occur later in the postoperative period. Postoperative CT would have been of high utility in confirming the diagnosis of POAP, but as radiological imaging is performed only when deemed necessary, a large proportion of this cohort had not undergone a postoperative CT. Owing to the retrospective nature of the study, misclassification bias during the collection and classification of complications is possible.

A recent consensus definition for post-pancreatectomy acute pancreatitis was released by the ISGPS^30^. Postoperative pancreatitis is defined as an acute inflammatory condition of the pancreatic remnant and the diagnosis is based on sustained elevation in plasma amylase levels (at least 48 hours postoperatively) with associated alterations in clinical course, and radiological alterations, such as parenchymal oedema or peripancreatic fluid collections. As stated previously, the lack of radiological images for many of the patients in this study cohort make it impossible to validate the new definition. This study points out that the inclusion of CRP in the new definition could be of use. Future studies might use CR-POAP as an outcome or investigate the inclusion of CRP in the newly proposed definition. CR-POAP shares most of the risk factors associated with CR-POPF, and, as an independent complication, CR-POAP may promote its onset. The updated alternative-FRS can be used to predict CR-POAP accurately.

## Supplementary Material

zrac012_Supplementary_Data

## Data Availability

Collected data will not be made available as the study permissions do not permit the sharing of individual patient data.
