# Supplementary material for: Prediction and consequences of postoperative pancreatitis after pancreaticoduodenectomy
Source: BJS Open. 2022 Apr 26;6(2):zrac012. doi: 10.1093/bjsopen/zrac012 (PMC9039121; doi:10.1093/bjsopen/zrac012)
Supplement: zrac012_Supplementary_Data [file zrac012_supplementary_data.zip › Supplementary_Table_1.docx]

| **Table S1.** Results of univariable and multivariable analyses on predictors for high postoperative morbidity (Comprehensive Complication Index ≥ 33.7) in 508 patients undergoing pancreaticoduodenectomy. | | | | | | | |
| --- | --- | --- | --- | --- | --- | --- | --- |
|  | | **Univariable analysis of risk factors for CCI ≥ 33.7** | | | **Multivariable analysis of risk factors for CCI ≥ 33.7** | | |
|  | | **CCI ≥ 33.7**  **(n = 141)** | **CCI < 33.7**  **(n = 367)** | **p-value** | **OR** | **CI 95%** | **p-value** |
| ***Continuous variables, median (IQR)*** | | | | | | | |
| **Age (years)** | | 68 (63 – 73) | 67 (61 – 73) | 0.316 |  |  |  |
| **BMI (kg/m^2^)** | | 26.86 (24.25 – 29.30) | 24.98 (22.32 – 27.34) | <0.001 | 1.07 | 1.02 – 1.13 | 0.011 |
|  |  |  |  |  | Unit of increase: kg/m^2^ |  |  |
| **Charlson index** | | 2 (2 – 3) | 2 (2 – 3) | 0.894 |  |  |  |
| **Estimated blood loss (ml)** | | 700 (400 – 1300) | 700 (450 – 1100) | 0.747 |  |  |  |
| **Main pancreatic duct diameter (mm)** | | 3 (2 – 5) | 4 (3 – 6) | <0.001 | 0.97 | 0.88 – 1.06 | 0.456 |
|  |  |  |  |  | Unit of increase: 1 mm |  |  |
| ***Categorical variables, n (%)*** | | | | | | | |
| **Sex** | |  |  |  |  |  |  |
|  | Male | 85 (60.3%) | 192 (52.3%) | 0.106 |  |  |  |
|  | Female | 56 (39.7%) | 175 (47.7%) |  |  |  |  |
| **Neoadjuvant therapy** | | 26 (18.4%) | 80 (21.8%) | 0.404 |  |  |  |
| **CR-POPF** | | 45 (31.9%) | 19 (5.2%) | <0.001 | 3.81 | 1.93 – 7.53 | <0.001 |
| **CR-POAP** | | 56 (39.7%) | 35 (9.5%) | <0.001 | 2.90 | 1.57 – 5.35 | <0.001 |
| **Preop. ERCP** | | 90 (63.8%) | 278 (76.0%) | 0.006 | 0.63 | 0.36 – 1.08 | 0.094 |
| **Venous resection** | | 33 (23.4%) | 93 (25.3%) | 0.651 |  |  |  |
| **Soft pancreatic texture** | | 92 (65.2%) | 167 (45.5%) | <0.001 | 1.26 | 0.70 – 2.24 | 0.440 |
| **Tumour histology** | |  |  |  |  |  |  |
|  | PDAC | 57 (40.4%) | 212 (58.1%) | <0.001 | 0.89 | 0.41 – 1.93 | 0.774 |
|  | IPMN or MCN | 12 (8.5%) | 23 (6.3%) | 0.371 |  |  |  |
|  | NET | 5 (3.5%) | 17 (4.6%) | 0.590 |  |  |  |
|  | Extrapancreatic malignancies (CholangioCA, PapillaryCA, DuodenalCA) | 42 (29.8%) | 72 (19.6%) | 0.014 | 1.25 | 0.53 – 2.92 | 0.611 |
|  | Other | 25 (17.7%) | 41 (11.2%) | 0.049 | 0.99 | 0.42 – 2.37 | 0.990 |

**Abbreviations:** POAP, Postoperative acute pancreatitis; CR, clinically relevant; BMI, Body mass index; ERCP, endoscopic retrograde pancreaticocholangiography; PDAC, Pancreatic ductal adenocarcinoma; IMPN intraductal mucinous papillary neoplasm; MCN, Mucinous cystic neoplasm; NET, Neuroendocrine tumour; CA, Carcinoma.
