# Supplementary figures and images for: Prediction and consequences of postoperative pancreatitis after pancreaticoduodenectomy
Source: BJS Open. 2022 Apr 26;6(2):zrac012. doi: 10.1093/bjsopen/zrac012 (PMC9039121; doi:10.1093/bjsopen/zrac012)

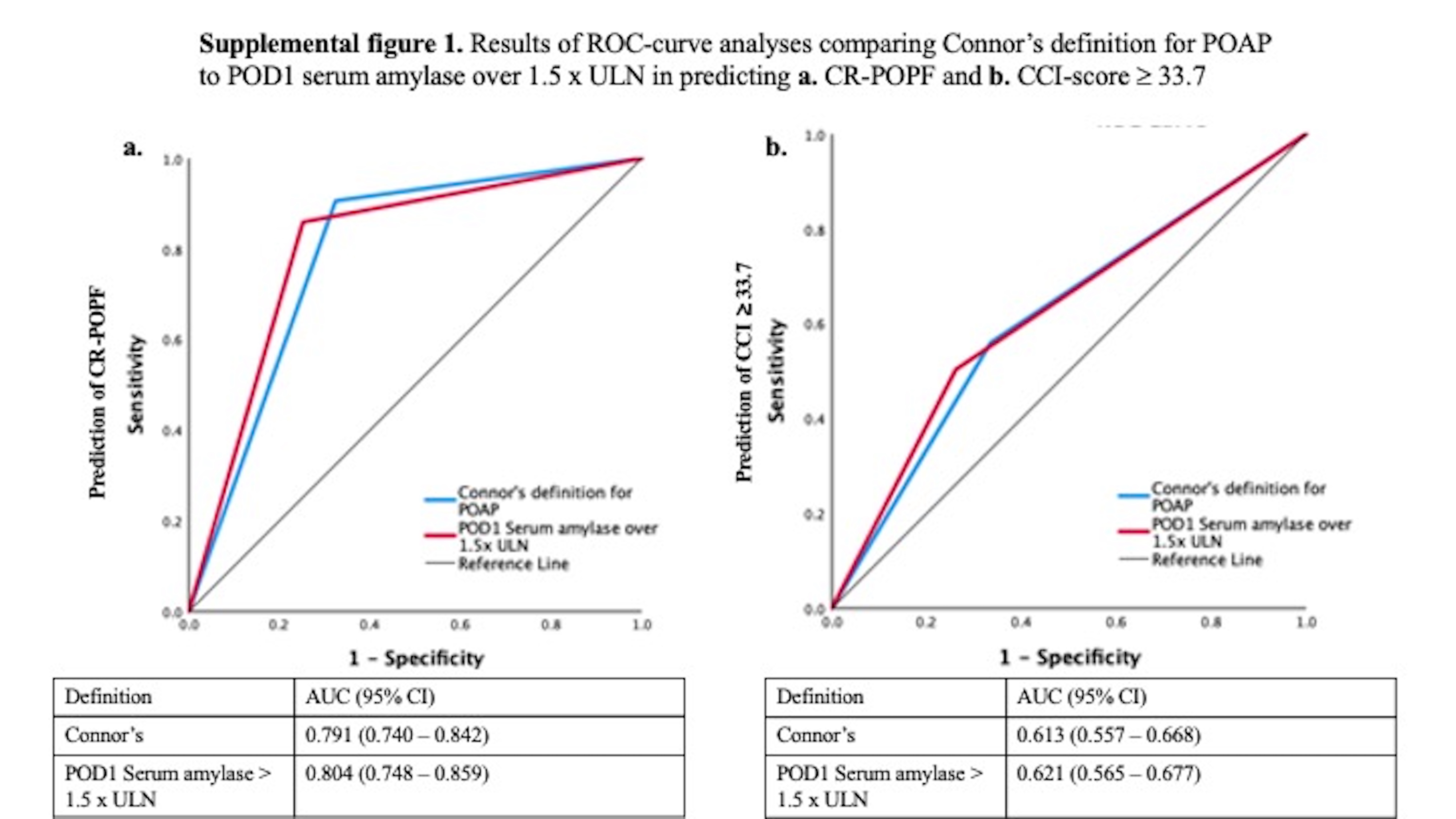

Supplement: zrac012_Supplementary_Data [file zrac012_supplementary_data.zip › Supplementary_Figure_1.jpg]
